# Impact of Chitosan and Benzalkonium Chloride as Final Irrigants on Fracture Resistance and Surface Roughness of Endodontically Treated Roots: An In Vitro Study

**DOI:** 10.1155/ijod/2387154

**Published:** 2026-07-21

**Authors:** Fereshteh Shafiei, Zahra Jowkar, Sepideh Eslamipanah

**Affiliations:** ^1^ Oral and Dental Disease Research Center, Department of Operative Dentistry, School of Dentistry, Shiraz University of Medical Sciences, Shiraz, Iran, sums.ac.ir; ^2^ Department of Operative Dentistry, School of Dentistry, Shiraz University of Medical Sciences, Shiraz, Iran, sums.ac.ir

**Keywords:** benzalkonium chloride, chitosan, fracture resistance, root canal irrigation, surface roughness

## Abstract

**Background:**

Chelating agents such as ethylenediaminetetraacetic acid (EDTA) effectively remove the smear layer but may weaken dentin and lack antimicrobial activity. Chitosan (CH), which has chelating and reported antibacterial properties, and benzalkonium chloride (BAC), an antimicrobial agent used after EDTA, may serve as alternative final irrigants. This study compared the effects of CH, BAC, and EDTA, alone or combined, on dentin surface roughness (SR) and fracture resistance (FR) of endodontically treated teeth.

**Methods:**

Ninety‐five extracted human mandibular first premolars with a single root canal were standardized to a 13 ± 1 mm root length and instrumented to ProTaper F4. For FR testing, 70 specimens were distributed into seven groups (*n* = 10): intact root (positive control), not‐obturated (negative control), normal saline, EDTA, EDTA /CH, EDTA/ BAC, and CH alone. Roots were obturated with AH Plus sealer and gutta‐percha, embedded in resin and loaded along the long axis until fracture. For SR assessment, 25 additional roots were sectioned longitudinally to obtain 50 specimens, which were then assigned to five groups based on the same final irrigation protocols used for FR (*n* = 10 per group). Atomic force microscopy (AFM) measured arithmetical mean SR (Ra, nm) at three midroot sites per specimen. Data analysis was performed (*p* = 0.05).

**Results:**

All obturated groups, including the normal saline group, showed significantly higher FR than the nonobturated group (*p* < 0.001). CH produced the highest FR, comparable to the intact root and significantly higher than EDTA, EDTA/CH, and normal saline groups (*p* < 0.05), but not significantly different from EDTA/BAC (*p* > 0.05). EDTA/CH and EDTA/BAC did not improve FR compared to EDTA alone (*p* > 0.05). Mean ± SD Ra values ranked from lowest to highest were: normal saline, CH, EDTA / BAC, EDTA / CH, and EDTA. All pairwise comparisons were statistically significant (*p* < 0.001).

**Conclusion:**

CH demonstrated the highest FR among the tested irrigation protocols while producing mild SR. EDTA showed intermediate FR values and was associated with the highest SR. The addition of BAC after EDTA resulted in relatively favorable FR with moderate SR. Notably, CH and EDTA/BAC exhibited comparable FR values, suggesting that both protocols may help maintain dentin mechanical integrity while providing acceptable surface characteristics.

## 1. Introduction

Successful root canal therapy depends on effective chemomechanical preparation, in which irrigating solutions play a critical role in disinfection and smear layer management [1]. Sodium hypochlorite (NaOCl) is widely used because of its strong antimicrobial activity and its ability to dissolve organic tissues. However, it is incapable of eliminating the inorganic portion of the smear layer generated during canal instrumentation [2, 3]. Consequently, a chelating agent is typically used after NaOCl to remove this layer [4]. Removal of the smear layer facilitates deeper penetration of intracanal medicaments and improves the adaptation and sealing ability of root canal sealers [5]. Among available chelators, ethylenediaminetetraacetic acid (EDTA) is the most frequently employed [4].

Nevertheless, NaOCl and EDTA can compromise root‐dentin integrity [6]. Exposure to NaOCl has been associated with reductions in dentin microhardness, elastic modulus, and overall mechanical strength. Its deproteinizing action may also create a weakened mineral framework that can decrease the fracture resistance (FR) of treated roots [7–9]. EDTA, on the other hand, promotes demineralization of peritubular and intertubular dentin, potentially leading to structural erosion and increased susceptibility to root fracture [10, 11]. When used sequentially with NaOCl, these effects may be amplified, resulting in greater deterioration of dentin mechanical properties [12]. Furthermore, EDTA‐induced calcium depletion exposes the collagen matrix, which may facilitate bacterial attachment [13].

Given the drawbacks of conventional irrigants, recent investigations have explored alternative agents that combine smear layer removal with antibacterial efficacy. Chitosan (CH), a natural polysaccharide composed of glucosamine and *N*‐acetylglucosamine units, is biodegradable, biocompatible, and bioadhesive, with a polycationic semicrystalline structure [14]. Owing to its interaction with negatively charged bacterial membranes, CH exhibits strong antibacterial activity and helps prevent bacterial recolonization [15]. In addition, CH has the capacity to chelate metal ions, allowing smear layer removal and dentinal tubule exposure while reportedly causing less erosion than EDTA [16].

Benzalkonium chloride (BAC) is another candidate for final irrigation. As a quaternary ammonium compound, it possesses surfactant properties and broad antimicrobial effects, and it has been shown to inhibit matrix metalloproteinases [17]. Previous studies have demonstrated that BAC application can reduce bacterial adhesion and biofilm formation on dentin surfaces [18]. Improved surface wettability following BAC use may also enhance sealer–dentin interactions.

Although CH has been reported to help preserve dentin microhardness and improve the bonding performance of AH Plus sealer, its effect on FR and dentin surface roughness (SR) of obturated roots has not been fully clarified [19]. Similarly, the influence of BAC on root strength and dentin surface characteristics when used as a final irrigant remains unclear. Therefore, this study was designed to compare CH and BAC with EDTA regarding their effects on the FR of endodontically treated roots and on dentin SR. The null hypothesis stated that no significant differences would exist among CH, BAC, and EDTA in their impact on FR and SR.

## 2. Methods

Ethical approval for this investigation was obtained from the Research and Ethics Committee of Shiraz University of Medical Sciences (Protocol Number IR.SUMS.DENTAL.REC.1403.006). The study procedures complied with the principles of the Declaration of Helsinki. Informed consent was secured from all patients for the use of their extracted teeth in this research.

Prior to the experiment, the operator performing the procedures was calibrated through repeated pilot procedures to ensure consistency in specimen preparation, irrigation protocol, and measurement procedures. Due to the nature of the irrigating solutions, the operator performing the irrigation procedures could not be blinded to the solution identity. However, the investigator responsible for the FR testing and the atomic force microscopy (AFM) SR measurements was blinded to the group allocation.

Ninety‐five extracted human mandibular first premolars with a single canal were collected from patients aged 17–25 years who underwent tooth extraction for orthodontic or periodontal indications. Periapical radiographs were obtained to confirm the presence of a single root canal. Teeth with more than one canal, root curvature greater than 5°, open apices, calcifications, resorptive defects, apical fractures, restorations, or visible structural damage were excluded and replaced. The external surfaces of all specimens were further inspected using a stereomicroscope (Carl Zeiss Inc., Oberkochen, Germany) at 20× magnification to ensure that no cracks, fractures, caries, or other defects were present.

The buccolingual and mesiodistal diameters at the cementoenamel junction (CEJ) were determined using a digital caliper (Pella Inc., Redding, CA, USA) with an accuracy of 0.01 mm. The mean buccolingual and mesiodistal measurements were 4.9 ± 0.5 mm and 4.2 ± 0.5 mm, respectively. Residual soft tissues were removed, after which the teeth were disinfected in 0.1% chloramine‐T solution for 48 h and subsequently stored in distilled water at 4°C for a maximum period of 1 month.

For standardization, the crowns were removed by sectioning the teeth perpendicular to their long axis just below the CEJ using a water‐cooled diamond disc (D&Z Diamant, Darmstadt, Germany), producing roots with a uniform length of 13 ± 1 mm.

### 2.1. Sample Size Calculation

The required sample size was determined using G^∗^Power 3.1 software (Heinrich Heine University, Düsseldorf, Germany) before the study began. The effect size was estimated from the FR means and standard deviations reported by Jowkar et al. [20], in which the reported values ranged from 636.90 to 1276.70 N. A one‐way fixed‐effects ANOVA model was used for the calculation based on the six instrumented experimental groups included in the FR analysis, excluding the intact positive control group. With a Type I error rate (*α*) of 0.05 and a statistical power of 80% (*β* = 0.20), the minimum required sample size was calculated to be four specimens per group. To enhance statistical robustness and compensate for potential specimen loss and biological variability, the final sample size was increased to 10 specimens per group. FR was considered the primary outcome variable for the power analysis, and the SR arm followed the same group allocation for methodological consistency.

### 2.2. FR Testing

A total of 70 roots were randomly selected for FR evaluation, with seven experimental groups (*n* = 10 per group). The methodology for root canal preparation and FR analysis was adapted from previous research [20]. After preparation and standardization of the specimens, the teeth were randomly allocated to the experimental groups using a computer‐generated randomization sequence. Each specimen was assigned a coded identification number to ensure unbiased group allocation.

### 2.3. Root Canal Instrumentation and Irrigation

Ten specimens were allocated to the positive control group (Group 1) and received no treatment. The remaining teeth were instrumented using the ProTaper NiTi rotary system (Dentsply Maillefer, Ballaigues, Switzerland) driven by an X‐Smart endodontic motor (Dentsply Sirona, Ballaigues, Switzerland) up to size F4. Each specimen was inspected both before and after instrumentation with a stereomicroscope (Carl Zeiss Inc., Oberkochen, Germany) at 20× magnification under fiber‐optic illumination (Zeiss OPMI Pico, Carl Zeiss, Oberkochen, Germany) to verify the absence of cracks or craze lines.

The working length was set at 1 mm short of the full root length. During canal preparation, irrigation was performed with 1 mL of 2.5% NaOCl (Sigma–Aldrich, St. Louis, MO, USA) after each instrument using a 27‐gauge irrigation needle (Endo‐Eze, Ultradent, South Jordan, UT, USA). To avoid apical extrusion of the solution, the root apices were sealed with utility wax (Cavex Set Up Wax, Cavex, Haarlem, Netherlands). Following completion of instrumentation, the canals were flushed with 5 mL of 2.5% NaOCl and then rinsed with 2 mL of normal saline.

Ten instrumented roots were allocated to the negative control group (Group 2) and were not obturated (not‐obturated group). The remaining samples were randomly divided into five experimental groups (*n* = 10), each undergoing final irrigation with 5 mL of the assigned solution:

• Group 3: Normal saline for 3 min (0.9% NaCl, Darou Pakhsh, Tehran, Iran)
• Group 4: 17% EDTA for 3 min (Pulpdent, Watertown, MA, USA)
• Group 5: 17% EDTA for 3 min followed by 0.2% CH for 1 min (Sigma–Aldrich, St. Louis, MO, USA)
• Group 6: 17% EDTA for 3 min followed by 1% BAC for 1 min (Merck KGaA, Darmstadt, Germany)
• Group 7: CH for 3 min (0.2% solution, Sigma–Aldrich, St. Louis, MO, USA)

A 0.2% (w/v) CH solution was formulated from high–molecular‐weight CH powder (degree of deacetylation 75%–85%, high viscosity; Sigma–Aldrich, St. Louis, MO, USA). The powder was dispersed in 1% (v/v) acetic acid and stirred magnetically at room temperature for ~2 h until a homogeneous solution was obtained. The pH was then adjusted to 3.2 with 1 M sodium hydroxide and confirmed using a calibrated digital pH meter. The solution was prepared immediately before use and kept in a light‐shielded container until application.

When two irrigants were used in sequence, an intermediate flushing step was carried out with 5 mL of distilled water (Milli‐Q, Merck Millipore, Germany) for 30 s to eliminate remnants of the first solution before introducing the second solution. Irrigation was performed with a 27‐gauge side‐vented needle (Endo‐Eze, Ultradent, South Jordan, UT, USA) inserted up to 1 mm short of the working length to minimize pressure and avoid apical extrusion.

After this rinse, the canals were lightly dried with sterile paper points (Meta Biomed, Cheongju, Korea) prior to applying the second irrigant. This approach allowed each solution to act on dentin independently without interaction with residual chemicals.

After completion of the final irrigation protocol, each canal was rinsed with 5 mL of distilled water (Milli‐Q, Merck Millipore, Germany) to eliminate the remaining irrigant residues. The canals were then dried with sterile paper points (Meta Biomed, Cheongju, Korea) and obturated using ProTaper F4 gutta‐percha cones (Dentsply Maillefer, Ballaigues, Switzerland) in combination with AH Plus sealer (Dentsply DeTrey, Konstanz, Germany) employing the single‐cone technique. The adequacy of the obturation was verified using periapical radiographs obtained with a digital imaging system (Digora Optime, Soredex, Tuusula, Finland).

To allow complete setting of the sealer, the specimens were wrapped in moist gauze (Nu Gauze, Johnson & Johnson, USA) and kept in sterile containers inside an incubator (Memmert, Schwabach, Germany) at 37 °C and 100% humidity for 1 week.

To simulate the periodontal ligament, a uniform 2 mm layer of C‐silicone impression material (Zetalabor, Zhermack SpA, Badia Polesine, Italy) was applied to the root surfaces up to 2 mm apical to the CEJ. The specimens were then embedded in self‐curing acrylic resin (Acropars, Marlik Co., Tehran, Iran), leaving ~10 mm of the apical portion of the root covered by the resin.

For FR testing, each specimen was positioned on the lower plate of a universal testing machine (Instron Z020, Zwick Roell, Ulm, Germany). A steel ball with a 5 mm diameter spherical tip was aligned with the center of the root canal orifice, and a compressive load was applied along the long axis of the root at a crosshead speed of 1 mm/min until fracture occurred. The maximum load at fracture was recorded in Newtons (N) using Bluehill software (Instron, Norwood, MA, USA).

### 2.4. SR Analysis

For SR evaluation using AFM, 25 teeth were randomly selected. Specimen preparation was performed according to a previously described protocol [21]. Each root was sectioned longitudinally into two parallel dentin slices using a slow‐speed cutting machine (Mecatome T201 A, Presi, Grenoble, France) under water cooling, resulting in 50 dentin halves and a total of 50 samples. The specimens were randomly allocated to five experimental groups (*n* = 10 per group). Each dentin half was treated and analyzed as an individual specimen.

To achieve a smooth surface, all dentin slices were sequentially polished using silicon carbide abrasive papers (400, 600, 800, and 1200 grit) under continuous distilled water irrigation to eliminate surface irregularities and scratches. For SR evaluation, 50 specimens were randomly allocated into five groups (*n* = 10 each). These groups (SR groups 1–5) were pretreated with the same solutions as groups 3–7 in the FR assessment. Each specimen was immersed in 5 mL of the designated solution at room temperature, with gentle agitation, for the same duration as the corresponding FR protocol in each group. The solutions were placed in sterile polypropylene microcentrifuge tubes (Eppendorf, Hamburg, Germany) and agitated at 100 rpm on a digital orbital shaker (IKA KS 130, IKA‐Werke, Staufen, Germany) to enhance the interaction with the dentin surface.

For experimental groups requiring sequential pretreatment with two different solutions, an intermediate rinse was performed between treatments to eliminate residual chemicals and prevent interactions. Each specimen was rinsed with 5 mL of distilled water (Milli‐Q, Merck Millipore, Germany) for 30 s using a precision pipette (Eppendorf, Hamburg, Germany) to ensure a controlled and thorough wash. Specimens were then carefully dried with lint‐free laboratory wipes (Kimwipes, Kimberly‐Clark, USA) before the application of the second solution.

After the exposure period, specimens were removed using sterile forceps (Hu‐Friedy, Chicago, IL, USA) and immediately subjected to a rinse with 5 mL of distilled water (Milli‐Q, Merck Millipore, Germany) for 30 s to eliminate any residual solution. They were then gently air‐dried for 10 s using an oil‐free air syringe positioned at a standardized distance of 10 mm to ensure consistent moisture removal.

### 2.5. AFM Assessment

SR was evaluated using AFM. For each specimen, three areas in the midroot region were scanned. Imaging was performed using a Naio AFM system (NanoSurf, Liestal, Switzerland) operating in tapping (intermittent‐contact) mode under ambient conditions.

A Tap150Al‐G sharp silicon cantilever (BudgetSensors, Sofia, Bulgaria) with an aluminum reflective coating was used, with a nominal resonance frequency of ~150–160 kHz and a nominal spring constant of about 5 N/m. Topographic images were obtained at a scan rate of 0.5 Hz over a 10 µm × 10 µm scan area with a resolution of 512 × 512 pixels. Image acquisition and analysis were performed using NanoSurf AFM Control Software (NanoSurf, Switzerland).

For each specimen, three separate fields of view were analyzed. The arithmetic mean SR (Ra, nm), defined as the average deviation of the surface profile from the mean line within the evaluation length, was calculated for each scanned area. The final Ra value for each specimen was determined by averaging the measurements obtained from the three scanned regions.

### 2.6. Statistical Analysis

Data were analyzed using SPSS software version 20.0 (IBM Corp., Armonk, NY, USA). For both FR and SR, normality was assessed using the Shapiro–Wilk test, and homogeneity of variances was evaluated using Levene’s test. Although the data for both outcomes were normally distributed (*p* > 0.05), the assumption of equal variances was violated (Levene’s test, *p* < 0.001). Therefore, Welch’s one‑way analysis of variance was used to compare groups for FR and SR. When significant differences were detected, pairwise comparisons were performed using Tamhane’s T2 post hoc test. The level of statistical significance was set at *p* < 0.05.

## 3. Results

The mean and standard deviation values for FR across the seven study groups are presented in Table 1. The Shapiro–Wilk test showed that the data were normally distributed in all groups (*p* > 0.05). However, Levene’s test demonstrated significant heterogeneity of variances among groups (*p* < 0.001). Therefore, Welch’s one‐way analysis of variance was used, which revealed a statistically significant difference in FR among the groups (*p* < 0.001). Pairwise comparisons were subsequently performed using Tamhane’s T2 post hoc test. All obturated groups, including the normal saline group, showed significantly higher FR compared to the nonobturated group (*p* < 0.001). The CH group demonstrated significantly greater FR than EDTA, EDTA/CH, and normal saline groups (*p* < 0.05). However, there was no significant difference between the CH and EDTA/BAC groups (*p* = 0.74). Additional irrigation with CH or BAC following EDTA did not result in a significant improvement compared to EDTA alone (*p* > 0.05). There was also no significant difference between the EDTA and normal saline groups. Both CH and EDTA/BAC groups exhibited FR values comparable to the intact root control group (*p* > 0.05).

**Table 1:** Fracture resistance (FR) values (mean ± SD) of the study groups and statistical comparisons.

Group	Irrigation/Condition	Mean ± SD (*N*)	Post hoc grouping
Group 1	Intact root (no preparation)	514.20 ± 97.42	A
Group 2	Instrumented, not‐obturated	216.80 ± 18.58	B
Group 3	Normal saline	319.60 ± 21.67	C
Group 4	EDTA	364.40 ± 74.44	CD
Group 5	EDTA/CH	310.70 ± 21.59	C
Group 6	EDTA/BAC	429.90 ± 68.51	AD
Group 7	CH	503.70 ± 94.63	A

*Note:* Different letters in the post hoc grouping column indicate statistically significant differences between groups (*p* < 0.05).

**Figure 1:**
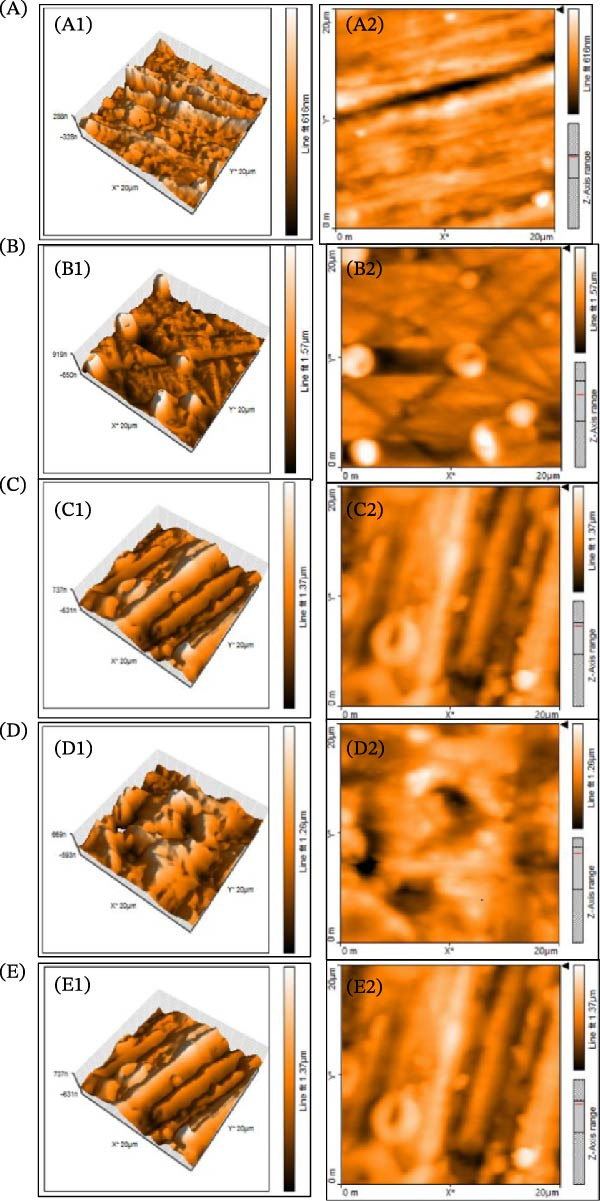
Representative atomic force microscopy (AFM) images of root canal dentin surfaces following different final irrigation protocols (10 µm × 10 µm scans). (A1) 3D topography of dentin treated with normal saline, showing a smooth and uniform surface with minimal irregularities. (A2) Corresponding 2D height map of the normal saline group, confirming low surface roughness. (B1) 3D topography after EDTA treatment, demonstrating pronounced surface irregularities with widened dentinal tubules and evident erosion. (B2) 2D height map of the EDTA group, illustrating high roughness values and significant surface variation. (C1) 3D topography following EDTA/CH irrigation, showing moderate irregularities with partial preservation of dentin structure. (C2) 2D height map of the EDTA/CH group, indicating reduced roughness compared to EDTA alone. (D1) 3D topography of dentin treated with EDTA/BAC, revealing a more homogeneous surface with less pronounced irregularities. (D2) 2D height map of the EDTA/BAC group, demonstrating intermediate roughness levels. (E1) 3D topography after CH treatment, showing a relatively smooth surface with minimal tubule enlargement and preserved morphology. (E2) 2D height map of the CH group, confirming low‐to‐moderate surface roughness. Abbreviations: AFM, atomic force microscopy; BAC, benzalkonium chloride; CH, chitosan; EDTA, ethylenediaminetetraacetic acid.

Representative AFM images of the root canal dentin surfaces for each irrigation group are shown in Figure 1. The mean and standard deviation values for SR in the five study groups are presented in Table 2. Post hoc tests revealed that normal saline produced the smoothest surface, whereas EDTA produced the roughest (both *p* < 0.001 vs. all). Adding CH or BAC after EDTA reduced SR significantly (*p* < 0.001), with EDTA/BAC smoother than EDTA/CH (*p* < 0.001). CH alone was smoother than EDTA/CH (*p* < 0.001). SR ranked from lowest to highest as normal saline, CH, EDTA/BAC, EDTA/CH, and EDTA. All pairwise differences were statistically significant.

**Table 2:** Mean surface roughness (Ra ± SD) of root canal dentin following different final irrigation protocols, as measured by atomic force microscopy (AFM).

Group	Irrigation protocol	Ra ± SD (nm)	Post hoc grouping
Group 1	Normal saline	75.31 ± 7.55	A
Group 2	EDTA	259.85 ± 9.93	B
Group 3	EDTA/CH	233.05 ± 15.82	C
Group 4	EDTA / BAC	181.14 ± 10.97	D
Group 5	CH	138.00 ± 12.91	E

*Note:* Different letters in the post hoc grouping column indicate statistically significant differences between groups (*p* < 0.05).

## 4. Discussion

The conventional irrigation sequence of NaOCl followed by EDTA effectively removes the smear layer but may also cause dentinal erosion [10, 11]. When NaOCl is applied after EDTA, it can intensify the chelating effect and lead to pronounced inter‐ and peritubular dentin damage [22]. Conversely, using NaOCl before EDTA may leave a residual hydroxyapatite layer that partially protects exposed collagen from further degradation [10]. Because omitting NaOCl as the final irrigant would eliminate its well‐known antimicrobial and antibiofilm benefits, the present study used CH and BAC after EDTA to preserve these advantages while potentially reducing dentin erosion. In addition, a separate group received CH alone, allowing it to function both as a chelating and antimicrobial agent. The results showed that the EDTA/CH and EDTA/BAC protocols did not significantly increase FR compared with EDTA alone, whereas CH used as a single irrigant produced significantly higher FR than EDTA. Therefore, the null hypothesis was only partially supported. Furthermore, all obturated and irrigated roots, including those rinsed with normal saline, exhibited significantly greater FR than the nonobturated specimens, highlighting the reinforcing effect of obturation with the AH Plus epoxy resin sealer.

Wright et al. [23] reported that sealer–dentin bonding may obscure the direct effects of irrigants on dentin mechanical properties, an influence that becomes more evident when canals are left unobturated. Nevertheless, many FR studies include obturation to better simulate clinical conditions and to assess the combined impact of irrigant‐induced dentin alterations and sealer adhesion on overall root strength [20, 24, 25]. This approach more closely reflects the clinical scenario, in which both dentin substrate changes and sealer bonding contribute to the mechanical behavior of the treated tooth. Although the relationship between bond strength and FR is not fully established, adequate adhesion between the sealer and root dentin may enhance the overall reinforcing effect [26].

Among the five obturated and irrigated groups, the FR of EDTA and EDTA/CH groups did not significantly differ from the normal saline group, while the CH group demonstrated markedly higher values than the EDTA, EDTA/CH, and normal saline groups. The FR of the EDTA/BAC group was comparable to that of the CH group. These findings indicate that smear layer removal alone may not determine FR. Other factors, including the potential adverse effects of EDTA on dentin mechanical properties, likely contribute. Chelating agents can alter dentin’s chemical composition and ultrastructure, which may reduce its resistance to fracture. Uzunoglu et al. [24] reported similar FR between EDTA (1‐min application) and normal saline groups, highlighting a limited effect of EDTA under short exposure conditions. In contrast, Turk et al. [25] found that roots treated with EDTA (1 min) followed by NaOCl exhibited significantly greater FR than those irrigated with normal saline alone. The effect of EDTA appears to be time‐dependent. Exposure times exceeding 3 min may result in collagen degradation and pronounced dentin erosion [1]. Although a 1 min exposure is often considered sufficient for smear layer removal, even short contact times have been associated with reduced dentin microhardness [22, 27]. However, this reduction does not necessarily translate into decreased FR of the entire root in the absence of obturation [27]. Prolonged exposure, such as 5 min, has been reported to significantly reduce FR without affecting flexural or cohesive strength [11]. In the present study, both EDTA and CH were applied for 3 min, based on previous evidence demonstrating effective smear layer removal and enhanced resin sealer infiltration at this duration [16]. CH applied for 3 min has been shown to open dentinal tubules with minimal peritubular erosion [16]. Although CH and EDTA exhibit comparable chelating capacity, CH does not adversely affect the dentin Ca/P ratio, unlike EDTA [28]. Furthermore, Antunes et al. [19] reported that both agents similarly reduced dentin microhardness while improving sealer penetration and bond strength.

Regarding the bond strength of mineral trioxide aggregate (MTA)–resin hybrid sealers, CH has demonstrated superior performance compared with EDTA [29]. In addition, CH has shown beneficial effects when used with AH Plus sealer, particularly in the coronal third of root dentin [30]. These findings are consistent with the significantly higher FR observed in the CH group compared with the EDTA group in the present study. The improved bond strength associated with CH irrigation may partly explain the higher FR values observed with CH. Its hydrophilic nature may enhance the wettability of hydrophobic resin sealers such as AH Plus on the dentin surface [31]. Moreover, CH can interact with collagen through covalent bonding, and its ability to bind to both the organic and inorganic components of dentin may contribute to improved mechanical properties [15]. A recent study reported that using CH as a final irrigant instead of EDTA did not reduce dentin microhardness; in fact, microhardness increased when CH was applied after EDTA [32]. In contrast, the present study did not demonstrate an additional benefit when CH (pH = 3.2) was used following EDTA. This may be attributed to the further chelating action of acidic CH on dentin that had already been demineralized by EDTA. Methodological differences may also explain the discrepancy. The previous study used CH nanoparticles at pH 5, and both EDTA and CH were applied for only 1 min [32]. The smaller particle size may have enhanced dentin penetration and facilitated interaction with the exposed collagen matrix [31, 32]. Moreover, another recent report showed that short‐term application of low‐molecular‐weight CH at pH 6.5 can chemically interact with dentin surfaces, regardless of prior EDTA treatment, and may provide additional antibacterial and antiproteolytic effects that could improve the durability of the resin–dentin bond [15]. The differences between those findings and the present results may therefore be related to the lower pH and high‐molecular weight of CH used in this study. High‐molecular‐weight CH may limit penetration into interfibrillar collagen and reduce effective chemical interaction. Future studies should investigate low‐molecular‐weight and nanoparticle forms of CH at less acidic pH levels, particularly in long‐term evaluations of dentin integrity and bond stability.

Another agent evaluated in this study, BAC, showed a moderately positive influence on FR. Although the difference in FR between the EDTA/BAC and EDTA groups was not statistically significant, the FR values of the EDTA/BAC group were comparable to those of the CH group and the intact root group. Previous studies have shown that EDTA irrigation may leave a residual layer of exposed collagen fibrils on the dentin surface, which can impair the ability of resin sealers to adequately wet and adhere to dentin [33]. BAC, acting as a cationic surfactant, may help remove these residual collagen fibrils and improve the wettability and penetration of the resin sealer into the dentin substrate.

SR of root canal dentin is an important factor because it influences both bacterial recolonization and sealer adaptation [21]. Clinically, SR has a dual effect on treatment outcomes. Excessive SR, often resulting from instrumentation and strong chelating agents such as EDTA, can increase bacterial adhesion and support biofilm persistence, raising the risk of reinfection [21]. In contrast, a moderate increase in surface texture may improve dentin wettability and enhance the penetration of resin sealers, promoting micromechanical interlocking and a more effective seal [21]. In the present study, the five final irrigation protocols produced a clear SR gradient. Normal saline generated the smoothest surface, followed by CH, EDTA/BAC, EDTA/CH, and EDTA alone. The high Ra values observed with EDTA are consistent with previous reports demonstrating that its strong calcium‐chelating action enlarges dentinal tubules and causes erosion of inter‐ and peritubular dentin, resulting in a mineral‐depleted and irregular surface [21, 34]. Applying CH after EDTA reduced, but did not fully reverse, the increase in SR, suggesting that its effects can only partially counteract EDTA‐induced demineralization [16, 35]. When BAC followed EDTA, SR decreased to an intermediate level, possibly because its surfactant properties reduce surface alterations while maintaining a microretentive structure [36]. CH used alone produced a relatively smooth surface, consistent with its milder chelating behavior and preferential interaction with the organic matrix, whereas normal saline largely preserved the baseline dentin topography [37].

When the two datasets are considered together, a possible group‐level relationship between SR and FR may be suggested. However, this interpretation should be made cautiously because the two outcomes were assessed using different specimen models and separate teeth. The FR specimens underwent canal instrumentation, sequential irrigation including NaOCl, and obturation, whereas the SR specimens consisted of polished dentin slabs exposed only to the final irrigants. Within these methodological limitations, groups showing greater surface alteration tended to present less favorable mechanical outcomes. EDTA alone produced the highest SR and was associated with relatively lower FR values, which is consistent with the known demineralizing effect of EDTA on dentin. In contrast, normal saline produced the smoothest surfaces but showed only moderate reinforcement compared with the nonobturated group, suggesting limited surface modification for effective sealer interactions. CH produced moderate SR while maintaining relatively high FR among the irrigated groups, with values comparable to the EDTA/BAC protocol. The intermediate SR observed after EDTA followed by BAC may indicate partial moderation of EDTA‐induced surface alterations; however, the FR values were not markedly different from those of EDTA alone. Because SR and FR were evaluated using separate experimental models, no direct statistical correlation could be established, and these observations should be interpreted as exploratory rather than causal relationships.

Some limitations must be acknowledged. Antimicrobial activity, bacterial reduction, and biofilm removal were not evaluated; therefore, although CH and BAC are known to have antibacterial properties, this study assessed only their effects on dentin SR and FR. Further research should examine their antimicrobial efficacy under similar conditions. FR and SR were analyzed using different models and separate specimens. FR samples underwent instrumentation, NaOCl irrigation, and obturation, whereas SR was measured on polished dentin slabs exposed only to the final irrigants. Thus, no direct correlation between SR and FR could be established. The absence of NaOCl in the SR model may also explain the relatively low SR in the normal saline group. BAC was tested only after EDTA and not as a standalone irrigant; therefore, its independent effects on SR and FR cannot be determined. Future studies should evaluate BAC alone. The loading protocol assessed mainly intrinsic root strength as force was applied vertically through the canal orifice without coronal restoration. Accordingly, the findings reflect root mechanical behavior rather than the FR of a fully restored tooth. Another potential limitation of the SR analysis is that two dentin halves obtained from the same tooth were included as separate specimens, which may introduce within‐tooth clustering. Therefore, the potential nonindependence of these measurements should be considered when interpreting the results. Future studies may benefit from the use of mixed‐effects or clustered statistical models to account for possible within‐tooth correlations.

Finally, this in vitro design does not replicate clinical conditions such as cyclic loading, temperature variation, enzymatic activity, or fatigue. Natural variability among extracted teeth and short‐term evaluation may also influence the results. AFM assessed only surface topography and did not evaluate subsurface or chemical changes. Future in vivo or ex vivo studies with dynamic loading are needed to evaluate long‐term effects. Clinical factors such as remaining tooth structure, coronal restoration, occlusion, and parafunction may also affect FR [38]. Further research should clarify the multifactorial causes of vertical root fracture and define optimal concentrations and application times for CH and BAC, including their effects on polymicrobial biofilms. Moreover, considering that variations in dental substrates and pretreatment protocols may influence the outcomes, the findings of the present study should be re‐evaluated in future investigations involving different dental substrates and pretreatment approaches [39, 40].

## 5. Conclusion

Among the tested irrigation protocols, CH demonstrated the highest FR while producing relatively mild SR. EDTA showed intermediate FR values that were not significantly different from the normal saline or EDTA/CH groups. The addition of BAC after EDTA resulted in relatively favorable FR with moderate SR, indicating that this protocol may help moderate EDTA‐induced surface alterations.

## Author Contributions

**Fereshteh Shafiei**: conceptualization, methodology, formal analysis, funding acquisition, resources, supervision, project administration, validation, writing – review and editing. **Zahra Jowkar**: conceptualization, methodology, investigation, data curation, formal analysis, visualization, supervision, writing – original draft, writing – review and editing. **Sepideh Eslamipanah**: investigation, data curation, methodology, validation, visualization, writing – original draft, writing – review and editing.

## Funding

This research was financially supported by Shiraz University of Medical Sciences, Shiraz, Iran (Grant 30056).

## Disclosure

All authors have read and approved the final version of the manuscript. Dr. Zahra Jowkar, as the corresponding author, had full access to all of the data in this study and takes complete responsibility for the integrity of the data and the accuracy of the data analysis.

## Ethics Statement

This study received ethical approval from the Research and Ethics Committee of Shiraz University of Medical Sciences (Protocol Number IR.SUMS.DENTAL.REC.1403.006) and adhered to the Declaration of Helsinki guidelines. Written informed consent was obtained from all patients whose extracted teeth were used in the study.

## Conflicts of Interest

The authors declare no conflicts of interest.

## Data Availability

The data that support the findings of this study are available from the corresponding author upon reasonable request.
